# Oral administration of *Lactiplantibacillus plantarum* GKK1 ameliorates atopic dermatitis in a mouse model

**DOI:** 10.3389/fmicb.2025.1566594

**Published:** 2025-06-03

**Authors:** Tzu-Chun Lin, Yu-Chieh Wu, You-Shan Tsai, Shih-Wei Lin, Chin-Chu Chen, Ming-Ju Chen, Yen-Po Chen

**Affiliations:** ^1^Biotech Research Institute, Grape King Bio Ltd., Taoyuan, Taiwan; ^2^Department of Animal Science and Technology, National Taiwan University, Taipei, Taiwan; ^3^Department of Food Science, Nutrition, and Nutraceutical Biotechnology, Shih Chien University, Taipei, Taiwan; ^4^Institute of Food Science and Technology, National Taiwan University, Taipei, Taiwan; ^5^Center for Biotechnology, National Taiwan University, Taipei, Taiwan; ^6^Department of Animal Science, National Chung Hsing University, Taichung, Taiwan; ^7^The iEGG and Animal Biotechnology Research Center, National Chung Hsing University, Taichung, Taiwan

**Keywords:** atopic dermatitis, *Lactiplantibacillus plantarum*, interleukin 2, interleukin 4, interleukin 5, interleukin 17, gut microbiota, short-chain fatty acids

## Abstract

**Background/objectives:**

Atopic dermatitis (AD) is a prevalent chronic skin condition, especially in young children, with rising incidence in developed countries. AD causes repeated scratching, and thus affecting quality of life. This study evaluated the effects and mechanisms of the probiotic *Lactiplantibacillus plantarum* GKK1 on AD symptoms in mice.

**Methods:**

Five-week-old BALB/c mice were divided into four groups (*n* = 8): control, AD, low-dose GKK1 (10^7^ CFU/day), and high-dose GKK1 (10^9^ CFU/day). GKK1 was intragastrically administered daily for 42 days. AD symptoms, skin histology, serum antibodies, inflammatory cytokine levels, gut microbiota composition, and short-chain fatty acids (SCFAs) in the intestines were assessed.

**Results:**

GKK1 showed improved skin appearance and reduced inflammation in AD mice, with high-dose GKK1 significantly reducing histological inflammation. The GKK1 treatment upregulated splenic interleukin (IL)-2, suppressed IL-4, IL-5 and IL-17 levels and increased intestinal *Lactobacillus* and *Bifidobacterium* spp., contributing to higher SCFAs production in intestine.

**Conclusion:**

Oral *L. plantarum* GKK1 effectively ameliorated AD symptoms and reduced inflammation in mice. Therefore, *L. plantarum* GKK1 may serve as a potential treatment for AD.

## 1 Introduction

Atopic dermatitis (AD) is a prevalent chronic inflammatory skin disorder which is caused by immunological imbalance, defective skin barriers, gene mutation, and environmental risk factors (Thomsen, 2014). People typically develop the symptoms of AD in childhood, and one fourth of them continue to suffer from AD in adulthood, which means the prevalence of AD is about 20% people during their lifetime, and the prevalence is on the rise (Spergel and Paller, 2003). Besides, people with AD will have an increased risk to develop asthma and allergic rhinitis (Serrano et al., 2019). The clinical manifestation was intensely pruritic and scaly papules. Repeated scratching constitutes disruption of sleep, loss of employment, and financial burden for their family, which imposes a great impact on the patient’s quality of life (Kemp, 2003).

AD primarily results from two reasons: skin barrier defects and immunological imbalance. Firstly, people with the mutation of filaggrin (FLG) gene, which can produce epidermal proteins responsible for intact skin barriers, lead to dry skin and higher risk of allergens and bacteria penetration to skin, consequently causing AD (De Benedetto et al., 2012; Xiao et al., 2021). Emollients are applied to prevent water loss of skin and soothe itching but cannot ultimately ameliorate AD. Secondly, AD is associated with imbalance of T helper (Th) cells type 1, 2, 17, and 22 and regulatory T (Treg) cells. Th2 differentiation dominates in patients with AD, and therefore production of cytokines IL-4, IL-5, IL-13, and IL-31 were induced, which then facilitate B cells to produce immunoglobulin E (IgE), and thus Th1 differentiation was suppressed (Eyerich and Novak, 2013). Medical treatment such as corticosteroids, Janus kinase inhibitors, calcineurin inhibitors, and anti-IL-31 monoclonal antibody are used to inhibit immune responses (Liberman et al., 2018; Reynolds and Al-Daraji, 2002; McInnes et al., 2019; Silverberg et al., 2020). However, due to side effects and usage restrictions on age, we attempt to search for alternative treatment for AD.

Probiotics play a protective role on the health of gut and skin. Probiotics help alter gut microbiota composition, and because of the association of gut and skin, the symptoms of AD are thus improved. In AD patients, *Staphylococcus aureus*, *Escherichia coli*, and *Clostridium difficile* are enriched, whereas *Lactobacillus, Bifidobacterium* and *Bacteroides* spp. are reduced in the gut microbiota, which thus results in Th1/Th2 imbalance (Lee et al., 2018). Probiotics can help regulate immune reaction by inducing cell activation signaling and suppressing proinflammatory responses (Ueno et al., 2011; Smits et al., 2005). For example, *L. plantarum* LM1004 significantly improved the AD-like symptoms by reducing Th2 and Th17 cell transcription factor levels and enhancing the transcription factor levels of Treg, Th1 cells, and FLG (Kim et al., 2019). Furthermore, probiotics contribute to maintain the intestinal epithelial integrity via elevated expression of tight junction protein (Hill et al., 2014). Probiotics metabolites such as short chain fatty acids can modulate immune system by inducing Treg cells and can inhibit survival of pathogens because of low pH (Salem et al., 2018). Research revealed that the severity of AD was negatively associated with the richness of butyrate-producing bacteria (Nylund et al., 2015). The underlying mechanisms of probiotics against AD include improvement of gut microbiota, regulation of immune responses, and protection of gut and skin barriers.

Probiotic *Lactiplantibacillus plantarum* (formerly *Lactobacillus plantarum*), with a long history in food industry, demonstrates various beneficial activities to humans, such as anti-inflammatory, antioxidative, and antimicrobial activities (Garcia-Gonzalez et al., 2021). Although previous studies have shown that *L. plantarum* can modulate Th1 and Th2 cytokine and alleviate clinical symptoms of AD, the alteration of gut microbiota and production of short chain fatty acids (SCFAs) after administration of *L. plantarum* did not mention before (Prameswari et al., 2017; Prakoeswa et al., 2022). The rise in prevalence of AD is concerning. Therefore, in this study, we investigated the effects of oral administration of *Lactiplantibacillus plantarum* GKK1, previously identified through in-vitro screening for its significant stimulation of TNF-*α* and IL-10, on the treatment of AD in mice.

## 2 Materials and methods

### 2.1 Preparation of *L. plantarum* GKK1

*L. plantarum* GKK1 was isolated from pickled chili peppers in Hualien, Taiwan, in 2015, and was provided by Biotechnology Center, Grape King Bio Ltd. (Taoyuan, Taiwan). GKK1 was first cultivated in MRS broth (Difco Laboratories, Detroit, MI, USA) with 1% (v/v) stock for 24 h at 37°C anaerobically. The phosphate-buffered saline (PBS) (Uni Biotech Co., Ltd., Ye-san-Gun, South Korea) was used to wash the pellet centrifuged. The pellet was then adjusted to concentrations of 10^7^ and 10^9^ CFU/mL by PBS, and the samples prepared once every 2 days was stored at 4°C for use.

### 2.2 AD protocol

**Figure 1:**
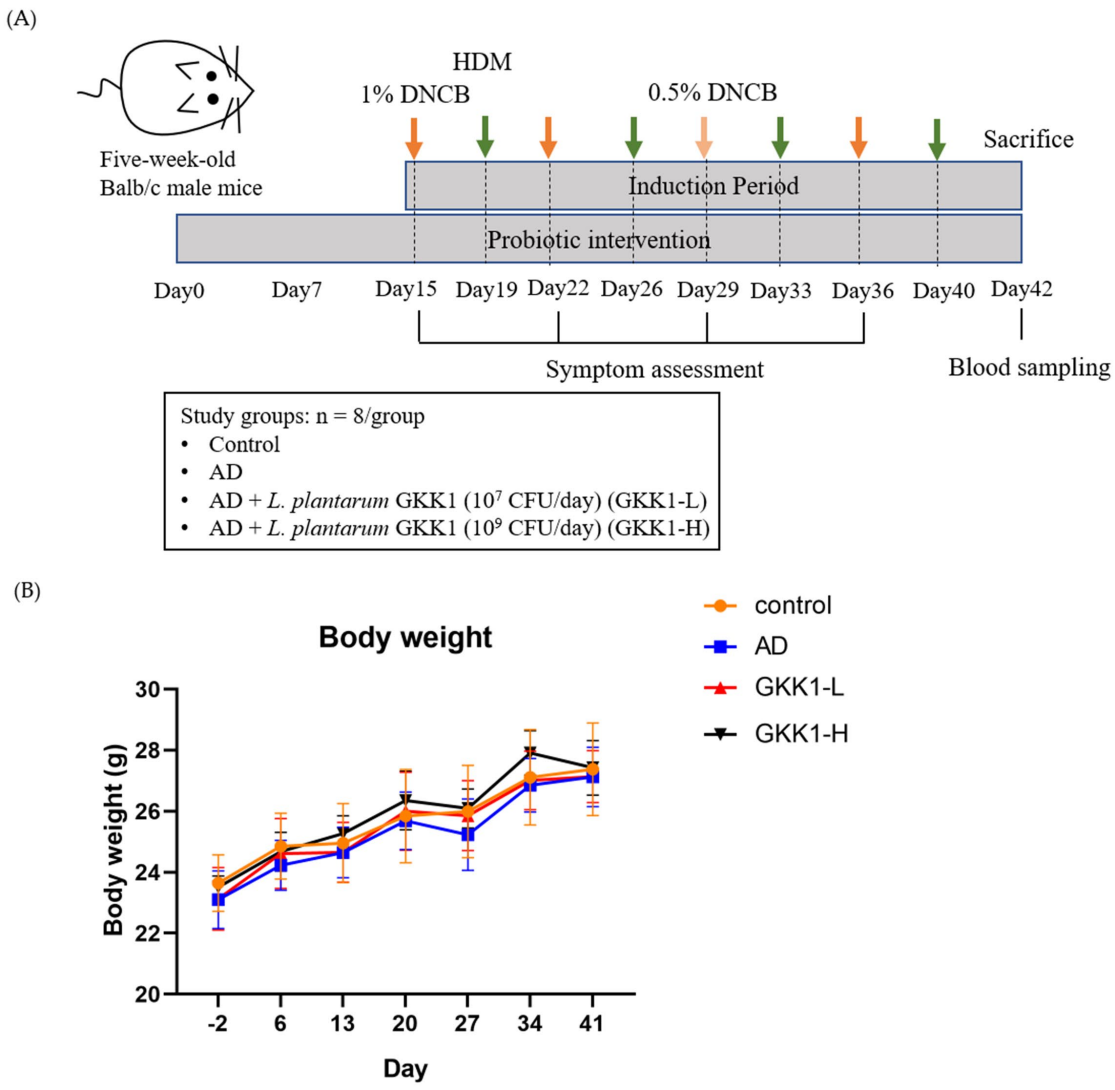
**(A)** The experimental design and **(B)** the body weight changes during the experiment period. Data are expressed as mean ± SD (*n* = 8 mice per group). Control: normal mouse control treated with phosphate-buffered saline (PBS), AD: 2,4-dinitrochlorbenzene (DNCB)-induced atopic dermatitis (AD) mouse treated with PBS, GKK1-L: DNCB-induced AD mouse treated with low-dosage *L. plantarum* GKK1; GKK1-H: DNCB-induced AD mouse treated with high-dosage *L. plantarum* GKK1.

Five-week-old male BALB/c mice were acquired from National Laboratory Animal Center (Taipei City, Taiwan) and were maintained in the National Taiwan University Animal Resource Center. In this study, 32 mice were divided into 4 groups (*n* = 8/group): control, AD, low-dosage *L. plantarum* GKK1 (GKK1-L) (10^7^ CFU/mouse/day), and high-dosage *L. plantarum* GKK1 (GKK1-H) (10^9^ CFU/mouse/day). The mice were first adapted for 1 week, and GKK1-L and GKK1-H groups were fed intragastrically with low and high dosage of GKK1 respectively, while 200 μL sterile PBS was supplied for control and AD groups daily. On day 14, the hair on the back was applied with hair removal cream (Church & Dwight Co., Inc., Ewing, NJ, USA) and shaved by a hair clipper (OSCAR 2000, Frega Enterprise Co., Ltd., Conquitlam, BC, Canada). Later, 1 cm^2^ surgical fabric adhesive tape (Symphon Medical Technology Co., Ltd., New Taipei City, Taiwan) was used to repeatedly strip and stick the mice’s back for five times to destroy skin stratum corneum. On days 15, 22, and 36, 20 μL acetone and olive oil (Sigma-Aldrich Inc., St. Louis, MO, USA) (acetone: olive oil = 1:3) with 1% 2,4-Dinitrochlorobenzene (DNCB, Sigma-Aldrich Inc.) was applied on the mice’s back in AD, GKK1-L, and GKK1-H groups; on day 29, 20 μL acetone and olive oil (acetone: olive = 1:3) with 0.5% DNCB was applied to all mice without control group. On days 19, 26, 33, and 40, 20 μL 0.5% Tween 20 in PBS with 10 mg/mL house dust mite extract (HDM, Stallergenes Greer Inc., MA, USA) was applied to AD and probiotic groups. As for control group, 20 μL acetone and olive oil (acetone: olive = 1:3) was given at the mentioned timepoint. Mice were sacrificed humanely by cervical dislocation under gas anesthesia by using isoflurane (Abbott Laboratories, Chicago, IL, USA)-soaked cotton on the 42th day. The experimental design was presented as Figure 1A. The study was registered on Institutional Animal Care and Use Committee (IACUC) of National Taiwan University under number 00038 in 2019.

### 2.3 Evaluation of symptoms of AD

The appearance of the skin in four groups of mice were observed before and after induction of AD. At the end of the study, the symptoms of AD were evaluated by the Yu et al.’s (2018) methods. Three categories, namely erythema/hemorrhage, scarring/dryness, and excoriation/erosion, were scored from 0 (mild) to 5 (severe) according to the area and the severity of the symptoms, and the total scores were 15.

### 2.4 Examination of histopathology and evaluation of dermatitis severity

After sacrifice, the skin and muscle tissue of the back (length 0.5 cm x width 1 cm) were removed and perfused in 10% formaldehyde in aqueous phosphate buffer (Mallinckrodt baker, Inc., Philipsburg, USA) for 24 h to fix the tissue. National Laboratory Animal Center was authorized for tissue slicing and staining. The tissues were embedded in paraffin and then sectioned. Later, the sections were stained with hematoxylin and eosin (hematoxylin and eosin stain, H&E stain) and observed by optical microscope. Further, the histological sections were evaluated by Duke Veterinary Clinic (Taipei, Taiwan) according to the scoring system (Fick et al., 2005) including epidermis and dermis. Thickness, acanthosis (thickening of the stratum spinosum, diffuse epidermal hyperplasia), hyperkeratosis (thickening of the stratum corneum), parakeratosis (retention of the keratinocyte nuclei in the stratum corneum), inflammation (intraepithelial inflammatory cell infiltration), erosion and ulceration (complete loss of epidermal cells) were determined in epidermis, whereas thickness, inflammation, necrosis, follicular keratosis, and fibrosis were determined in dermis. Considering ratio of area of symptoms to that of the sections, every symptom was graded from 0 (within normal limits) to 5 (severe). As for thickness score, it was ranged from −4 (severely thin) to +4 (severely thick) compared with the thickness of control group.

### 2.5 Analysis of serum concentration of IgE and IgG1/IgG2a ratio

Before euthanasia, the blood was collected from orbital sinus to blood collection tubes (BD Biosciences, Franklin Lakes, NJ, USA). The serum was obtained after centrifugation at 4,600 rpm for 5 min at 4°C and was then stored at −80°C for antibody analysis. The serum concentration of IgE, IgG1, and IgG2a were measured by enzyme-linked immunosorbent assay (ELISA) according to manufacturer’s instruction (Thermo Fisher Scientific Inc., Waltham, MA, USA).

### 2.6 Splenocyte cytokine profile analysis after HDM stimulation

After sacrificing the mice, spleens were placed in sterile RPMI 1640 medium and transferred to a sterile workbench. They were ground using a sterile syringe plunger on a cell strainer and washed with 10 mL RPMI 1640 medium. The filtered solution was centrifuged at 300 × *g* for 5 min, and the supernatant was discarded. Red blood cell lysis buffer was added, mixed, and centrifuged again. The residue was washed with PBS, centrifuged, and resuspended in RPMI 1640 medium with 10% FBS, penicillin, and streptomycin. Cells were adjusted to 5 × 10^7^ cells/mL, centrifuged, and resuspended in RPMI 1640 medium. The cell suspension was added to a 24-well plate with HDM and co-cultured for 48 h. The supernatant was collected and stored at −80°C for cytokine analysis. Cytokines IL-2, IL-4, IL-5, IL-10, and IL-17 in the serum samples were analyzed by ELISA. The steps could be followed according to the booklets of mouse cytokine kits (R&D Systems, Inc., Mckinley, MN, USA), and the absorbance of the samples was detected by spectrum 450 and 540 nm using spectrophotometer (Biotek Instruments Inc., Winooski, VY, USA).

### 2.7 Analysis of short chain fatty acids in guts

The analysis method was modified by Torii et al. (2010). After sacrifice, the samples in fresh ceca, large intestines, and feces were collected in the tubes, then 70% ethanol was added with 9 times as the weight of the samples. After homogenization, the samples were centrifuged at 13,200 rpm for 10 min at 4°C, and the supernatant was stored at −80°C for further analysis. Standards of acetic acid, propionic acid, butyric acid were obtained from Sigma-Aldrich. Working solutions of 0.0625, 0.125, 0.25, 0.5, and 1 mM were prepared by diluting standards with 50% methanol, and 2-ethylbutyric acid (2-BA, Sigma-Aldrich Inc.) was used as internal standard adjusted to concentration of 800 μM with 100% ethanol.

Each sample was undergone derivatization process according to Torii et al.’s (2010) method. The sample (150 μL) was mixed with internal standard (50 μL) and 150 μL each of Reagent 1 [3% pyridine (Aldrich Chemical Co., Inc., Milwaukee, WI, USA) ethanol (Honeywell International Inc.) solution], Reagent 2 [1-(3-dimethylaminopropyl)-3-ethylcarbodiimide hydrochloride, EDC-HCl (Tokyo Chemical Industry Co., Ltd., Tokyo, Japan) ethanol solution], and Reagent 3 [2-nitrophenylhydrazine hydrochloride, 2-NPH-HCl (Tokyo Chemical Industry Co., Ltd.) ethanol solution] in a 15 mL centrifuge tube, reacted at 60°C for 20 min, and then mixed with 200 μL of Reagent 4 [15% potassium hydroxide solution (Sigma-Aldrich Inc.), methanol (Aencore Chemical Co., Ltd., Surry Hills, NSW, Australia) solution], and reacted at 60°C for 20 min. After cooling, the mixture was shaken with 1.5 mL of Reagent 5 [0.5 M phosphoric acid, H_3_PO_4_ aqueous solution (Aldrich Chemical Co., Inc.)] and 2 mL of diethyl ether (Honeywell International Inc.) and stayed still for 3 min and then centrifuged at 1,500 rpm for 3 min. The obtained ether layer was shaken with 2.0 mL of water for 3 min and centrifuged. The ether layer was transferred to a 2 mL Eppendorf tube, and ether was eliminated in a hood. The obtained fatty acid hydrazide was dissolved in 200 μL of 100% methanol and filtered through a 0.22 μm filter, and the sample was subjected to a high-performance liquid chromatography system (PU-2089 Quaternary HPLC Pump, JASCO International Co., Ltd., Tokyo, Japan). The chromatographic column used is a C18 column (ReproSil 100 C18 5 μM, 250 × 4.6 mm, Dr. Maisch GmbH, Ammerbuch, Germany). The mobile phase was comprised of acetonitrile (J.T. Baker Chemical Company, Phillipsburg, NJ, USA)–methanol–water (30:16:54), adjusted to a pH of 4.5 with 0.1% trichloroacetic acid (Sigma-Aldrich Inc.).

### 2.8 Microbiota analysis

The samples in fresh large intestines were first mixed with ddH_2_O (9 times the weight of the sample) and 1 mm grinding balls before vortexing at 1,500 rpm for 1 min. Then, an aliquot of 200 μL suspension was collected and mixed with 1 mL sterile PBS thoroughly in the homogeneous tube, followed by a centrifuge step (12,000 rpm, 5 min, 4°C). Later, the supernatant was removed, and the samples were stored at −20°C for further DNA extraction via the process (Neumann et al., 1992). The target bacteria genus *Lactobacillus* spp. and *Bifidobacterium* spp. were also undergone DNA extraction and determined the count by hemocytometer (SLGC, Tokyo, Japan). Then, real-time polymerase chain reaction [qPCR, StepOne Real-Time PCR System (Thermo Fisher Scientific Inc.)] was performed with serial 100-, 10-, and 1-fold dilutions of the target strains to obtain the standard curves of cycle threshold (Ct) value versus count (log CFU/g), and the populations of the target strains in the samples were calculated by Ct value from the standard curves. An Optical 8 Strip QPCR Tube (Bioman Scientific Co., Ltd., New Taipei City, Taiwan) contained 2 μL of DNA sample, 5 μL of KAPA SYBR FAST qPCR Master Mix (2×) (Kapa Biosystems Ltd., Salt River, Cape Town, South Africa), 0.2 μL of 10 μM forward primer, 0.2 μL of 10 μM reverse primer, and 2.6 μL of sterilized ddH_2_O. The annealing condition at qPCR stage was 55°C for *Lactobacillus* and 60°C for *Bifidobacterium*. The primer sets used in this study were referenced from Lubbs et al. (2009). For *Lactobacillus* spp., the forward primer was GGAAACAG(A/G)TGCTAATACCG (Lab-0159) and the reverse primer was ATCGTATTACCGCGGCTGCTGGCA (Univ-0515). For *Bifidobacterium* spp., the forward primer was CTCCTGGAAACGGGTGG (g-Bifid-F) and the reverse primer was GGTGTTCTTCCCGATATCTACA (g-Bifid-R).

### 2.9 Statistical analysis

The values were presented as the mean ± standard deviation (SD). One-way analysis of variance (ANOVA) using Duncan’s post-hoc test was conducted to evaluate the differences between every group. The value of statistical significance was set at *p* < 0.05. The analysis was performed using the SPSS statistical software package.

## 3 Results

### 3.1 *L. plantarum* GKK1 improved AD-like symptoms in mice

**Figure 2:**
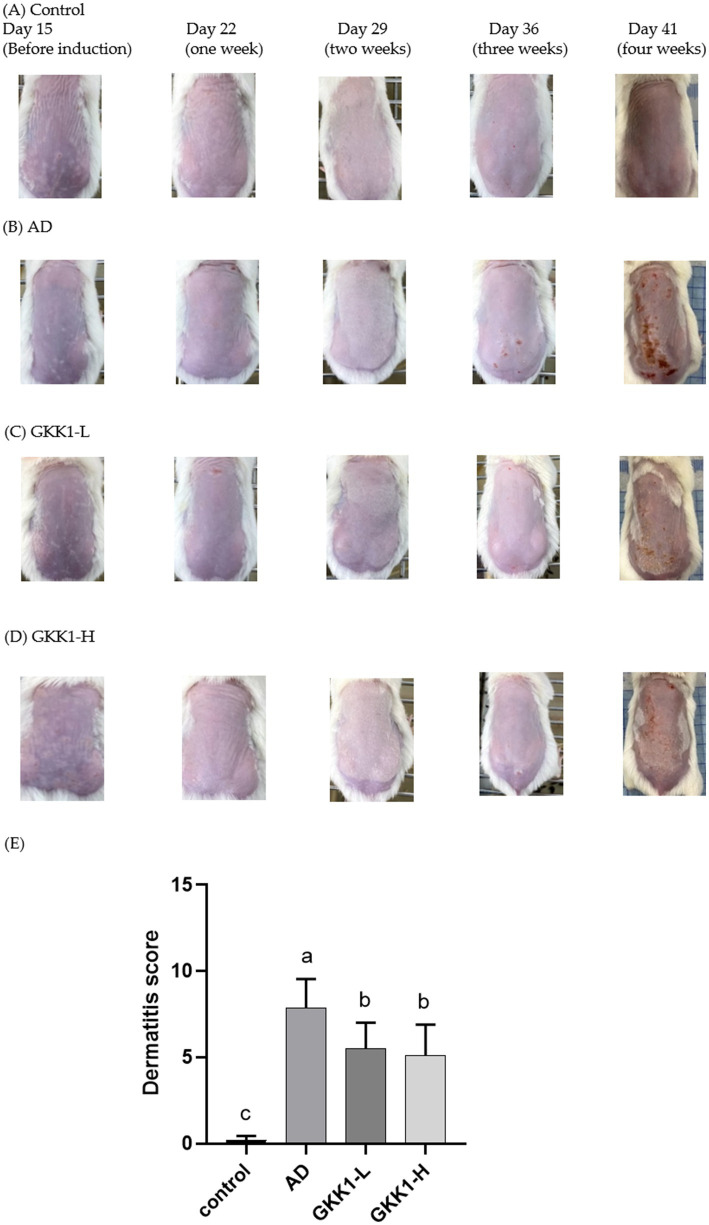
The appearance of skin in **(A)** control, **(B)** AD, **(C)** GKK1-L, **(D)** GKK1-H groups observed during the study, and **(E)** the scores of the clinical signs in four groups at the end of the study. Data are expressed as mean ± SD (*n* = 8 mice per group). Different letters (a, b, c) indicates significant difference at *p* < 0.05 as determined by one-way ANOVA. Control: normal mouse control treated with phosphate-buffered saline (PBS), AD: 2,4-dinitrochlorbenzene (DNCB)-induced atopic dermatitis (AD) mouse treated with PBS, GKK1-L: DNCB-induced AD mouse treated with low-dosage *L. plantarum* GKK1; GKK1-H: DNCB-induced AD mouse treated with high-dosage *L. plantarum* GKK1.

There were no significant differences on the body weight among experimental groups (Figure 1B), indicating that treatment of *L. plantarum* GKK1 did not affect the growth of mice. The clinical signs of AD in four groups were observed weekly (Figures 2A–D). After induction of AD (on day 36) by topical application of DNCB and HDM, the AD group exhibited severe erythema and hemorrhage. However, symptoms improved with oral administration of *L. plantarum* GKK1. Specifically, skin symptoms were scored at the end of the study (Figure 2E). The AD group had a score of 7.88 ± 1.66, significantly higher than the control group’s score of 0.19 ± 0.26 (*p* < 0.05), indicating successful induction of skin lesions. The scores of GKK1-L and GKK1-H groups were 5.50 ± 1.51 and 4.81 ± 1.71, respectively, both significantly lower than the AD group (*p* < 0.05).

### 3.2 *L. plantarum* GKK1 recovered the histology of epidermis and dermis in AD mice

**Figure 3:**
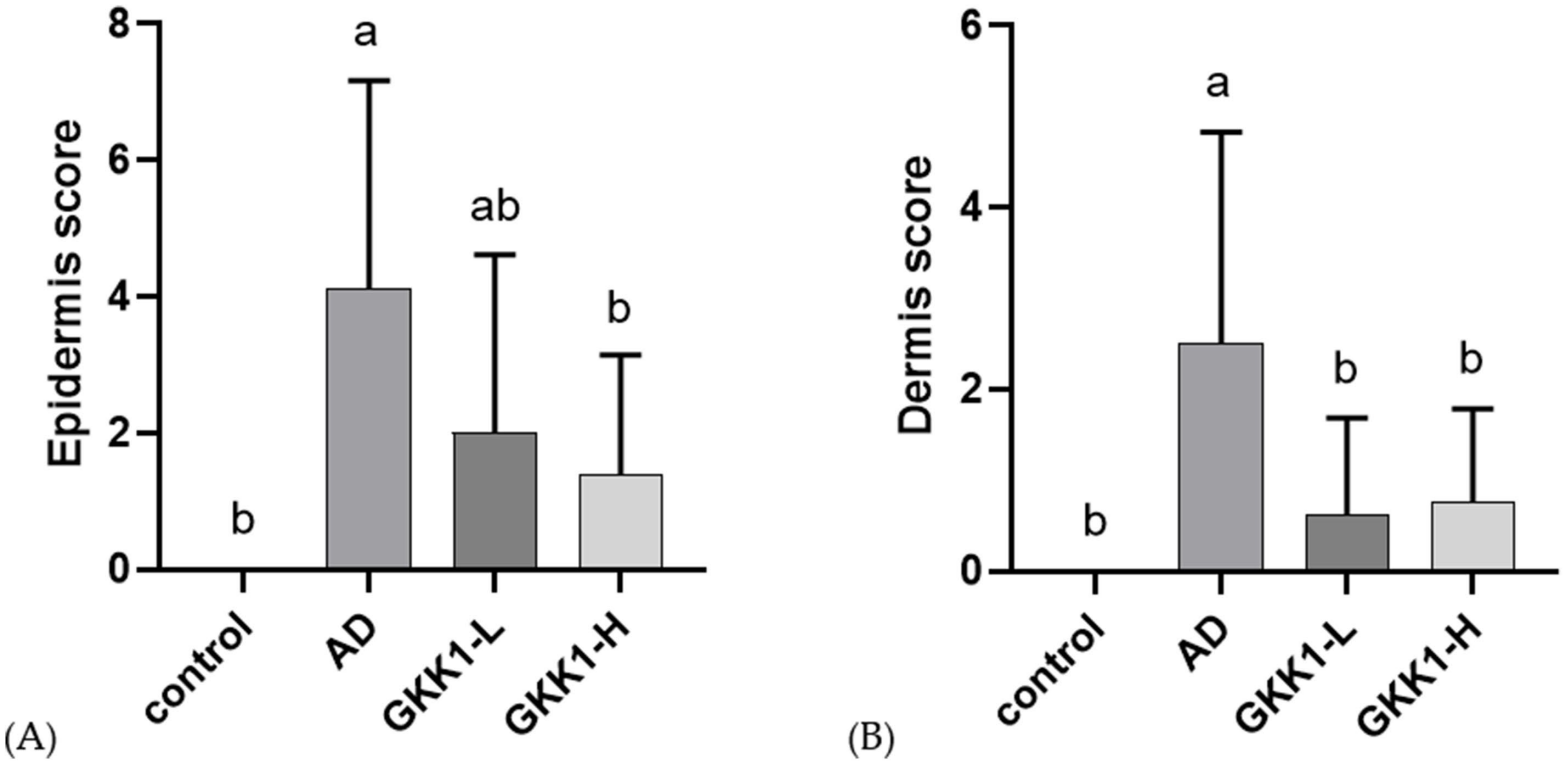
Quantitative evaluation of **(A)** epidermis, **(B)** dermis analysis of mouse skin tissue in different experimental groups. Data are expressed as mean ± SD (*n* = 8 mice per group). Different letters (a, b) indicates significant difference at *p* < 0.05 as determined by one-way ANOVA. Control: normal mouse control treated with phosphate-buffered saline (PBS), AD: 2,4-dinitrochlorbenzene (DNCB)-induced atopic dermatitis (AD) mouse treated with PBS, GKK1-L: DNCB-induced AD mouse treated with low-dosage *L. plantarum* GKK1; GKK1-H: DNCB-induced AD mouse treated with high-dosage *L. plantarum* GKK1.

The skin sections stained with hematoxylin and eosin were examined by the veterinarian. In epidermis analysis (Figure 3A), the score in the AD group was significantly higher than that of control group (*p* < 0.05), while the score in GKK1-L group was lower than that of AD group. The GKK1-H group had a significantly lower score than the AD group (*p* < 0.05). Regarding the dermis examination (Figure 3B), the AD group’s score was significantly higher than the control group’s (*p* < 0.05). Furthermore, there were significant differences in dermis scores between AD and two probiotics groups (*p* < 0.05).

### 3.3 *L. plantarum* GKK1 downregulated serum concentration of IgE and IgG1/IgG2a ratio in AD mice

**Figure 4:**
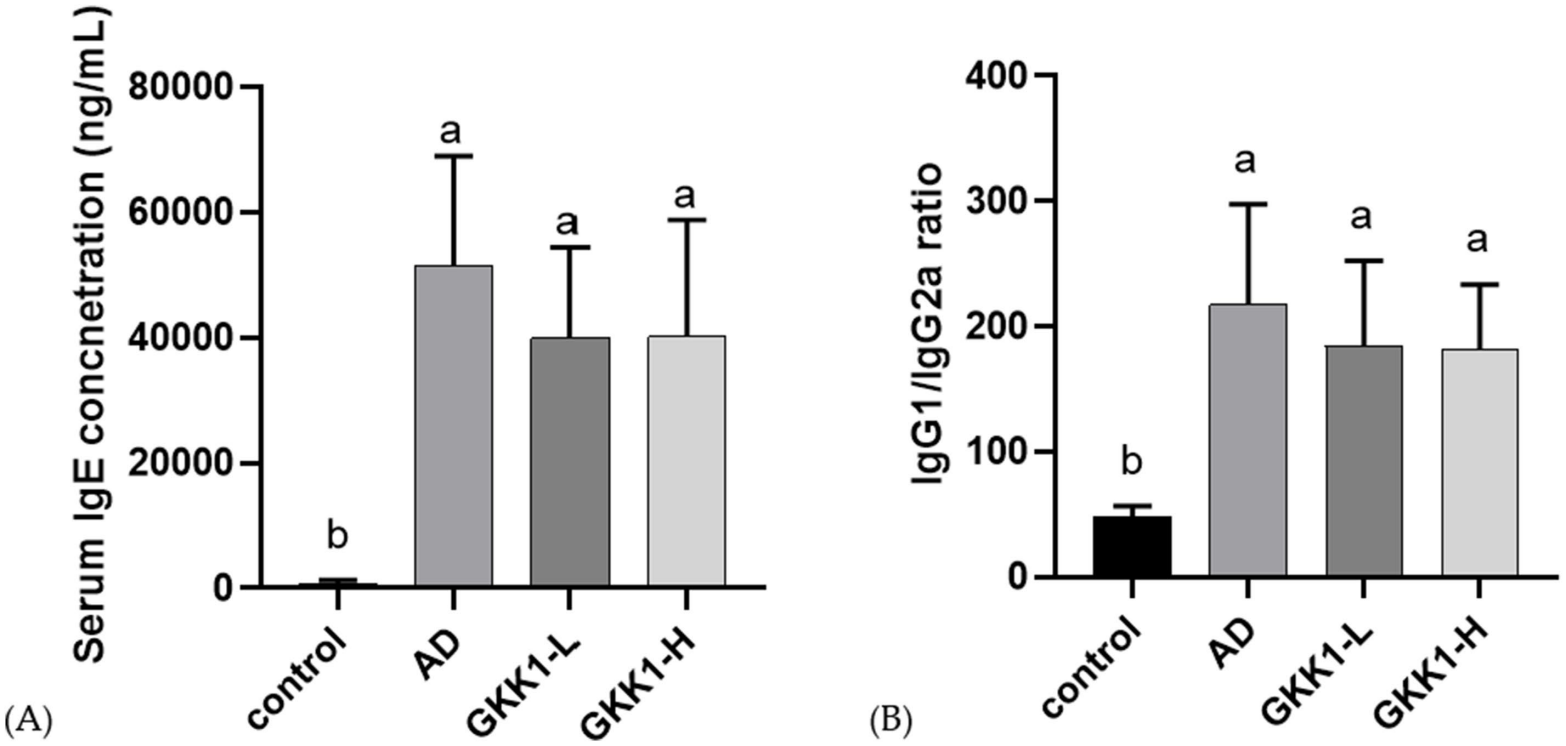
Serum antibody analysis in control, AD, GKK1-L, GKK1-H groups. **(A)** Serum IgE concentration **(B)** IgG1/IgG2a ratio. Data are expressed as mean ± SD (*n* = 8 mice per group). Different letters (a, b) indicates significant difference at *p* < 0.05 as determined by one-way ANOVA. Control: normal mouse control treated with phosphate-buffered saline (PBS), AD: 2,4-dinitrochlorbenzene (DNCB)-induced atopic dermatitis (AD) mouse treated with PBS, GKK1-L: DNCB-induced AD mouse treated with low-dosage *L. plantarum* GKK1; GKK1-H: DNCB-induced AD mouse treated with high-dosage *L. plantarum* GKK1.

The serum collected on day 42 was analyzed for the concentration of IgE, IgG1, and IgG2a. The results showed no significant differences in IgE levels between the probiotics groups (GKK1-L and GKK1-H) and the AD group (Figure 4A). However, IgE concentration tended to decrease following the administration of *L. plantarum* GKK1. As shown in Figure 4B, no statistically significant differences in the IgG1/IgG2a ratio were observed between the probiotic groups (GKK1-L and GKK1-H) and the AD group, though the ratio was lower in both probiotics’ groups compared to the AD group.

### 3.4 *L. plantarum* GKK1 raised the levels of cytokines IL-2 and lowered IL-4, IL-5 and IL-17 in spleen of AD mice

**Figure 5:**
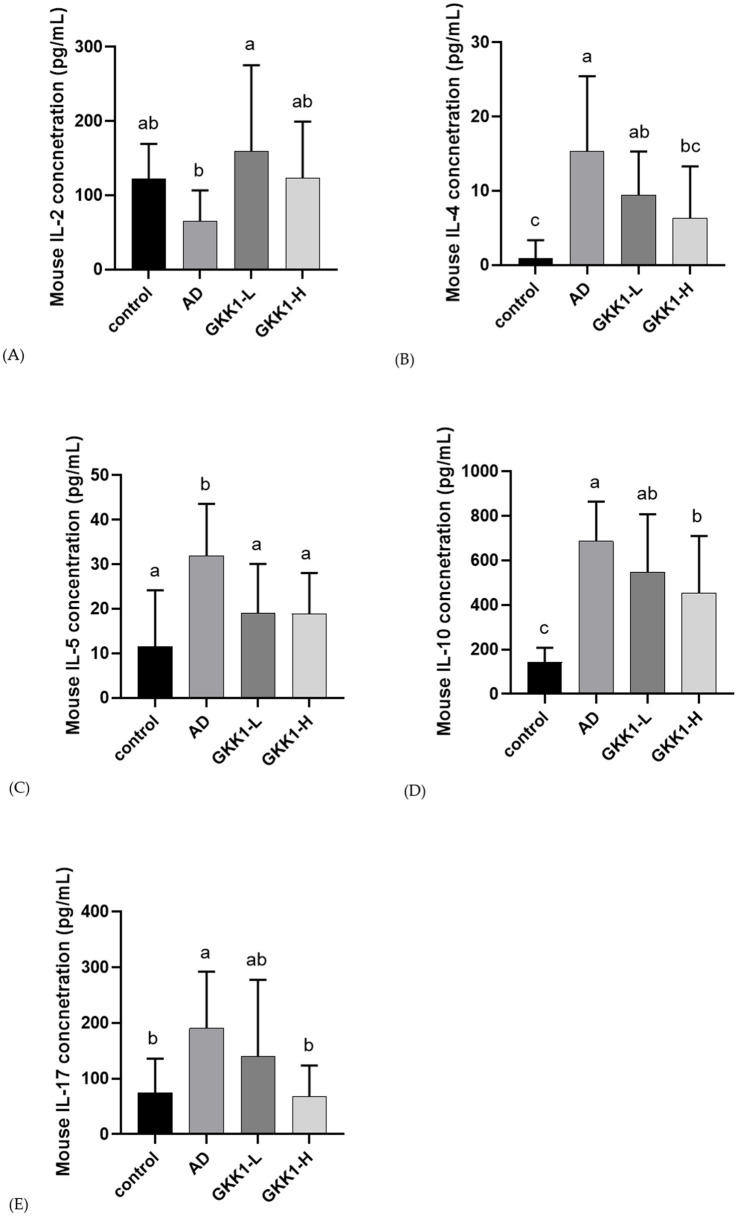
Splenic cytokine analysis in different experiment groups. **(A)** IL-2 **(B)** IL-4 **(C)** IL-5 **(D)** IL-10 **(E)** IL-17. Data are expressed as mean ± SD (*n* = 8 mice per group). Different letters (a, b, c) indicates significant difference at *p* < 0.05 as determined by one-way ANOVA. Control: normal mouse control treated with phosphate-buffered saline (PBS), AD: 2,4-dinitrochlorbenzene (DNCB)-induced atopic dermatitis (AD) mouse treated with PBS, GKK1-L: DNCB-induced AD mouse treated with low-dosage *L. plantarum* GKK1; GKK1-H: DNCB-induced AD mouse treated with high-dosage *L. plantarum* GKK1.

After 42 days of probiotics intervention, the concentrations of IL-4, IL-10 and IL-17 significant decreased in GKK1-H group compared to the AD group (*p* < 0.05), as shown in Figures 5B,D,E. Additionally, the GKK1-L group significantly increased IL-2 levels (Figure 5A), while the GKK1-L and GKK1-H groups had significantly lowered IL-5 levels compared to the AD group (Figure 5C).

### 3.5 Effects of *L. plantarum* GKK1 administration on *Lactobacillus* and *Bifidobacterium* in gut of AD mice

**Figure 6:**
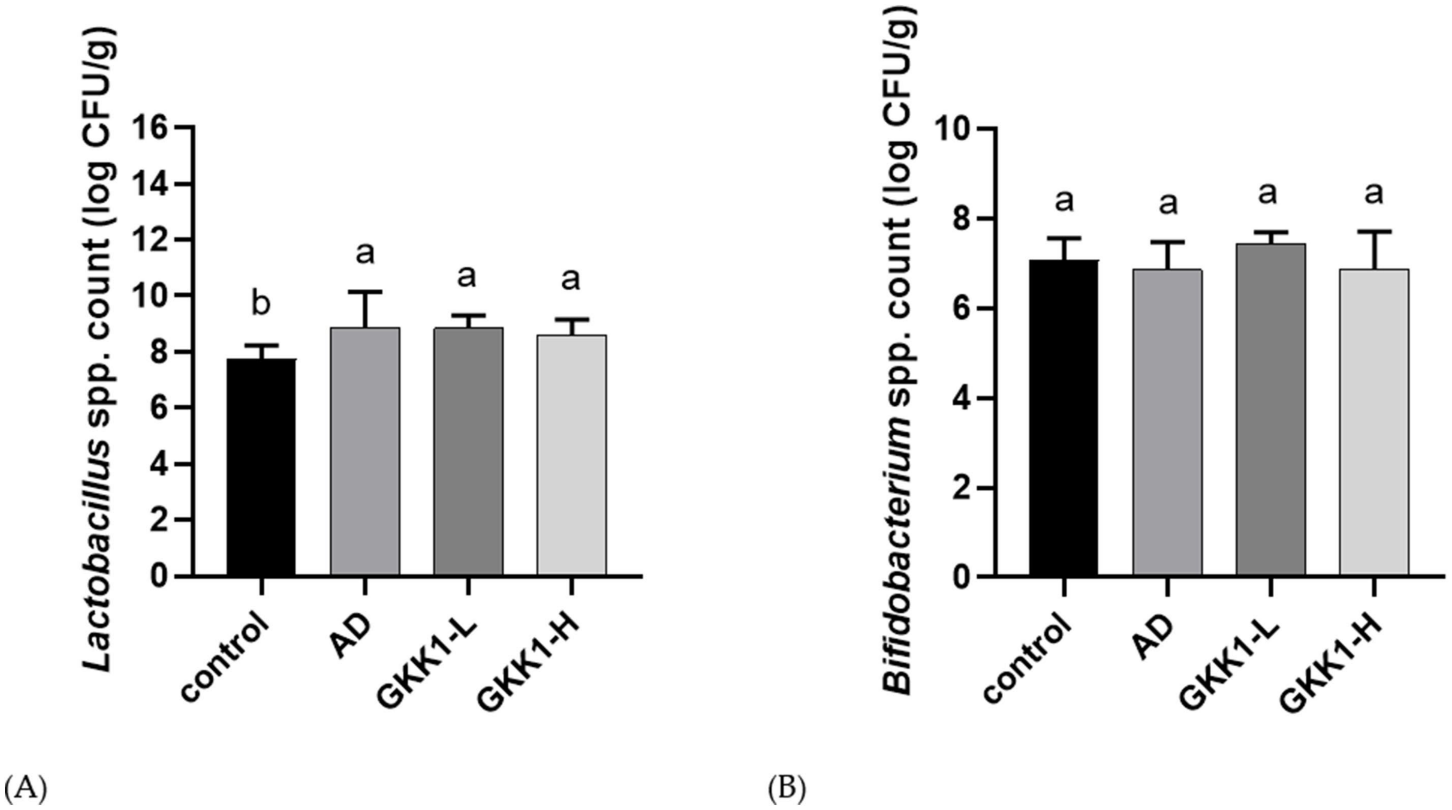
Quantification of intestinal **(A)** *Lactobacillus* spp. **(B)** *Bifidobacterium* spp. in different experiment groups. Data are expressed as mean ± SD (*n* = 8 mice per group). Different letters (a, b) indicates significant difference at *p* < 0.05 as determined by one-way ANOVA. Control: normal mouse control treated with phosphate-buffered saline (PBS), AD: 2,4-dinitrochlorbenzene (DNCB)-induced atopic dermatitis (AD) mouse treated with PBS, GKK1-L: DNCB-induced AD mouse treated with low-dosage *L. plantarum* GKK1; GKK1-H: DNCB-induced AD mouse treated with high-dosage *L. plantarum* GKK1.

The results of the *Lactobacillus* and *Bifidobacterium* content in the guts were shown in Figure 6. *Lactobacillus* levels were significantly increased in the AD, GKK1-L, and GKK1-H groups compared to the control group (*p* < 0.05). Additionally, *Bifidobacterium* content was higher in the GKK1-L group compared to the AD group without significant difference.

### 3.6 *L. plantarum* GKK1 increased acetic acid and propionic acids in gut of AD mice

**Figure 7:**
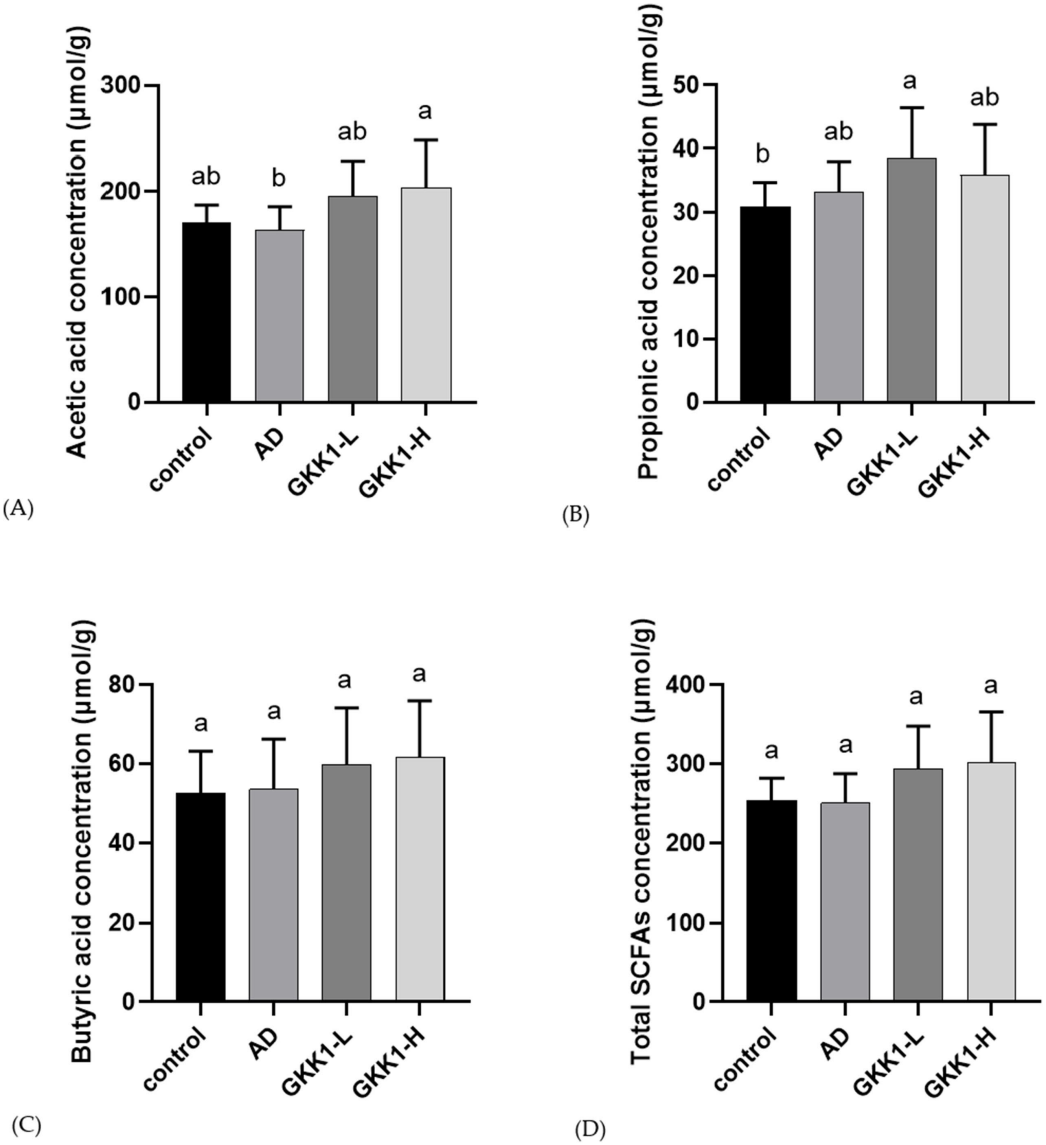
Quantification of short-chain fatty acids in mouse cecum in different experiment groups. **(A)** Acetic acid **(B)** Propionic acid **(C)** Butyric acid **(D)** Total SCFAs. Data are expressed as mean ± SD (*n* = 8 mice per group). Different letters (a, b) indicates significant difference at *p* < 0.05 as determined by one-way ANOVA. Control: normal mouse control treated with phosphate-buffered saline (PBS), AD: 2,4-dinitrochlorbenzene (DNCB)-induced atopic dermatitis (AD) mouse treated with PBS, GKK1-L: DNCB-induced AD mouse treated with low-dosage *L. plantarum* GKK1; GKK1-H: DNCB-induced AD mouse treated with high-dosage *L. plantarum* GKK1; SCFAs: short-chain fatty acids.

Concentrations of intestinal acetic acid in the GKK1-H group was significantly higher than that in the AD group (*p* < 0.05) (Figure 7A). The concentration of propionic acid in GKK1-L group was significantly higher than control group (*p* < 0.05) (Figure 7B). The levels of butyric acid and total SCFAs did not reach statistical significances (Figures 7C,D). However, total SCFAs showed an upward trend in the GKK1-H group compared to the control group (*p* < 0.1).

## 4 Discussion

In the present study, we demonstrated that administration of *L. plantarum* GKK1 showed the AD preventing effect in HDM-extract induced AD mice. The clinical symptoms of AD include erythema, edema, dryness/scarring, and excoriation/erosion (Lee et al., 2010), and the severity of AD was measured according to the symptoms. The BALB/c mice developed AD-like lesions after application of DNCB and HDM-extract, consistent with previous finding (Yoon et al., 2015). The consumption of both low and high dosages of GKK1 significantly improved the symptoms of AD. Skin biopsies from the AD group revealed skin lesions characterized by thickened epidermis and dermis, along with lymphocytic infiltrates in the dermis. Additionally, severe pyknosis of nuclei in the epidermis, indicative of cell apoptosis in AD, was observed (Hou et al., 2016). In line with previous studies, the thickness and inflammation severity of the epidermis and dermis were measured and scored in this AD mouse model. Consistent with prior findings, the AD group exhibited higher scores compared to the control group (Jang et al., 2018). In our study, the high dosage of GKK1 resulted in a significant improvement in both epidermal and dermal conditions, demonstrating a dose-effect relationship.

The mouse with AD exhibited upregulated serum IgE levels and an increase IgG1 to IgG2a ratio, which are markers of AD progression (Lee et al., 2010). Following intervention with probiotics GKK1, the elevation of serum IgE and the IgG1 to IgG2a ratio were suppressed without significant differences. During AD development, CD4^+^ T cells are activated and subdivided into Th1, Th2, Th17, and Treg cells (Su et al., 2017). IL-2, a cytokine produced by Th1 cells, is responsible for activating Treg cells and inhibiting Th17 cells (Hoyer et al., 2008). After administering probiotics to mice with DNCB-induced AD for 42 days, splenic IL-2 production significantly increased in GKK1-L group. Th2 cells, B cells, and mast cells are the major cell types involved in AD. Furthermore, Th2 cytokines, including IL-4 and IL-5, are associated with IgE secretion and eosinophil development, survival, and proliferation, and their up-regulation has been observed in patients with AD (Hoyer et al., 2008). In this study, IL-4 and IL-5 production in the spleen of AD mice treated with probiotics was suppressed, and high dosage of GKK1 showed better effect than low dosage of GKK1. These results indicate that probiotics GKK1 are beneficial for elevating Th1 cytokines and reducing Th2 cytokines.

IL-10 is an essential cytokine produced by Treg cells for suppression of immune inflammation (Ouyang et al., 2011). The level of IL-10 has been observed to inversely correlate with the severity of AD. Our investigation showed that the splenic IL-10 levels was higher in the AD group compared to the control group, consistent with previous studies (Laouini et al., 2003). In other studies, IL-10 production significantly increased after probiotics treatment (Lim et al., 2017; Pessi et al., 2000). However, in our investigation, IL-10 secretion was downregulated after probiotics intervention compared to the AD group. We believe that the timing of IL-10 evaluation could affect the results. It’s possible that IL-10 levels increased immediately after GKK1 treatment to control immune responses, but we only analyzed cytokines on day 42. We speculate that by day 42, the symptoms of AD had improved, leading to decreased IL-10 levels. Regarding IL-17, produced by Th17 cells, it plays a crucial role in the immune defense against allergies (Sugaya, 2020). Previous studies have shown that Th17 cell proportions and IL-17 levels are elevated in the peripheral blood of AD patients (Koga et al., 2008). With the intervention of a high dose of GKK1, IL-17 levels significantly decreased, indicating that GKK1 can modulate the Th17 immune system.

There is a cross-talk between gut microbiota and development of AD (Stefanovic et al., 2020). Hence, we further investigated whether gut microbial composition was altered after the treatment of probiotic GKK1. The results revealed that the probiotic GKK1 increased the content of *Lactobacillus* and *Bifidobacterium* in the gut. Previous studies have shown that the patients receiving both *Lactobacillus* and *Bifidobacterium*, or even either of them, experienced significant improvement in AD symptoms (Iemoli et al., 2012; Klewicka et al., 2011). These findings support our results, suggesting that an increase in *Lactobacillus* and *Bifidobacterium* in the gut can alleviate AD clinical manifestation. Treatment with the probiotic GKK1 contributed to altering the gut microbial environment, leading to increased production of SCFAs, including acetic and propionic acids, through bacterial fermentation of indigestible carbohydrates. SCFAs in the gut can inhibit the growth of other pathogens by lowering pH and can also enhance immune cell levels, such as Treg cells (Smith et al., 2013; Duncan et al., 2009).

Based on the results in this research, we speculate that the probiotic *L. plantarum* GKK1 may have the similar therapeutic effects on AD in humans, so the clinical trial of AD patients can be further conducted to verify in the future.

## 5 Conclusion

Our study highlights the therapeutic potential of *L. plantarum* GKK1 in alleviating symptoms of atopic dermatitis (AD) through its immunomodulatory effects, regulation of cytokine profiles, ability to modulate gut microbiota composition and SCFA productions. While further investigations are warranted to fully elucidate its mechanisms of action, *L. plantarum* GKK1 emerges as a compelling candidate for therapeutic intervention in AD.

## Data Availability

The raw data supporting the conclusions of this article will be made available by the authors, without undue reservation.
